# Minocycline Effects on IL-6 Concentration in Macrophage and Microglial Cells in a Rat Model of Neuropathic Pain

**DOI:** 10.22045/ibj.2016.04

**Published:** 2016-11

**Authors:** Taraneh Moini-Zanjani, Seyed-Nasser Ostad, Farzaneh Labibi, Haleh Ameli, Nariman Mosaffa, Masoumeh Sabetkasaei

**Affiliations:** 1 Shahid Beheshti University of Medical Sciences, Department of Pharmacology and Neuroscience Research Center, Tehran, Iran; 2 Tehran University of Medical Sciences, faculty of Pharmacy, Tehran, Iran; 3 Shahid Beheshti University of Medical Sciences, Department of Immunology, Tehran, Iran

**Keywords:** Minocycline, Interleukin-6, Macrophages, Microglia

## Abstract

**Background:**

Evidence indicates that neuropathic pain pathogenesis is not confined to changes in the activity of neuronal systems but involves interactions between neurons, inflammatory immune and immune-like glial cells. Substances released from immune cells during inflammation play an important role in development and maintenance of neuropathic pain. It has been found that minocycline suppresses the development of neuropathic pain. Here, we evaluated the analgesic effect of minocycline in a chronic constriction injury (CCI) model of neuropathic pain in rat and assessed IL-6 concentration from cultured macrophage and microglia cells.

**Methods:**

Male Wistar rat (n=6, 150-200 g) were divided into three different groups: 1) CCI+vehicle, 2) sham+vehicle, and 3) CCI+drug. Minocycline (10, 20, and 40 mg/kg) was injected one hour before surgery and continued daily to day 14 post ligation. Von Frey filaments and acetone, as pain behavioral tests, were used for mechanical allodynia and cold allodynia, respectively. Experiments were performed on day 0 (before surgery) and days 1, 3, 5, 7, 10, and 14 post -injury. At day 14, rats were killed and monocyte-derived macrophage from right ventricle and microglia from lumbar part of the spinal cord were isolated and cultured in RPMI and Leibovitz’s media, respectively. IL-6 concentration was evaluated in cell culture supernatant after 24 h.

**Results:**

Minocycline (10, 20, and 40 mg/kg) attenuated pain behavior, and a decrease in IL-6 concentration was observed in immune cells compared to CCI vehicle-treated animals.

**Conclusion:**

Minocycline reduced pain behavior and decreased IL-6 concentration in macrophage and microglial cells.

## INTRODUCTION

Evidence for the function of immune system in neuropathic pain is increasing[1]. Neuropathic pain is often results from peripheral and central nervous system (CNS) injuries, and it can be characterized by hyperalgesia and allodynia[2]. Immune activation takes place whenever there are peripheral nerves or associated tissues damage. Different lines of evidence suggest that neuroinflammation, mediated by the interaction between immune cells and neurons, plays an important role in pathological pain[3,4]. In both traumatic and inflammatory models, the key immune cells involved in the level of the peripheral nerve are neutrophils and macrophages. These cells are attracted by chemokines and recruited into the affected area from the general circulation, along with a host of locally resident cells[5]. It is also clear from studies of traumatic and inflammatory neuropathies that immune activation is not restricted to the periphery, rather, spinal cord immune involvement occurs in the form of glial activation[6,7]. Accumulating documents over last several years indicate that microglial cells are involved in the pathogenesis of neuropathic pain[2]. Pro-inflammatory cytokines are responsible for the early immune response through communicating between immune cells. However, the effects of pro-inflammatory cytokines can directly increase nerve excitability, damage myelin and alter the blood-nerve barrier. The involvement of pro-inflammatory cytokines in the creation and maintenance of neuropathic pain is mostly mediated by tumor necrosis factor (TNF), IL-1 and IL-6 effects[6,8]. Animal models of neuropathic pain have been developed to understand the pathogenesis of pain. Most models involve injury to the part of the sciatic nerve[5]. In this regard, chronic constriction injury (CCI) can evoke a substantial local inflammatory reaction[9]. Some studies have attempted to find substances that reduce the pro-inflammatory effects of cytokine. Among them, pentoxifylline and minocycline can decrease microglial and astrocyte activation by inhibition of cytokines, thus depressing neuropathic pain development[10,11].

In the present study, we evaluated the analgesic effects of minocycline used pre-emptively in a CCI model of neuropathic pain in rat, and the levels of secreted IL-6 from macrophage and microglial cells were measured.

## MATERIALS AND METHODS

Male Wistar rats (n=6) weighing 150-200 g were used. All animals were maintained under the same conditions (22±1°C, 60% relative humidity, 12 h light/dark cycle, food, and water *ad libitum*). Animals were accustomed to the housing facilities for at least one week before any treatment. All experiments followed the ethical guidelines of the International Association for the Study of Pain in animals[12].

### Chronic constriction injury to the sciatic nerve

Rats were anesthetized with 60 mg/kg ketamine and 10 mg/kg xylazine. After exposing sciatic nerve, four chromic catgut (4-0) ligations were tied loosely with ∼1 mm spacing, proximally to the sciatic trifurcation. Finally, the skin and muscle were sewed using silk sutures (4-0). In sham-operated animals, the same surgery was performed but the nerve was not ligated[9].

### Pharmacological studies

Minocycline (Sigma-Aldrich, USA) at 10, 20, and 40 mg/kg was dissolved in normal saline (0.09%) and was injected intraperitoneally one hour before surgery and then once daily (between 8:00 A.M. and 10:00 A.M.) for the following 14 days. In addition, allodynia-like behaviors were assessed. On day 14, animals were scarified. Macrophages and microglial cells were isolated and cultivated. Subsequent IL-6 analysis from the cell culture supernatant was performed after 24 h.

### Drug administration

Animals were divided randomly into three experimental groups: 1) CCI vehicle-treated, 2) sham-operated and 3) CCI drug-treated. Normal saline (0.09% NaCl) was injected to CCI and sham-operated animals as a vehicle. All behavioral tests were recorded on days 0 (control day), before surgery and 1, 3, 5, 7, 10, and 14 post ligation. Experiments were conducted between 12:00-14:00 P.M and the order of pain testing was mechanical and cold allodynia, respectively. The interval between each test was 30 min. On day 14, rats were euthanized by CO_2_ asphyxiation, and blood macrophages were collected from left ventricle under sterile condition. For the cultivation of microglial cells, rats were rapidly guillotined, and the lumbar part of the spinal cord was obtained for microglia isolation and cytokine analysis.

### Mechanical allodynia

Mechanical hypersensitivity was assessed by a set of calibrated von Frey filaments using a previous method[13]. Animals were placed in boxes on an elevated metal mesh floor and allowed 30 min for habituation before testing. A series of von Frey filaments with logarithmically incrementing stiffness (Stoelting, USA) was applied perpendicular to the mid-planter region of the hind paw. The mean paw withdrawal threshold was defined as the minimum gram strength producing two sequential responses with 3-min intervals between them (withdrawal from pressure)[13].

### Cold allodynia

Acetone test was used as a model of “cold allodynia”. In this method, rats were placed in boxes as described previously, and the heel of the paw was touched with an acetone bubble; it was formed at the end of a piece of small polyethylene tubing, connected to a syringe. The test was applied five times with one-minutes intervals. The response was calculated as the percent of paw withdrawal frequency using the following equation: number of paw withdrawals/five trials)⊆100[10].

### Macrophage isolation

Rats were euthanized by inhalation of CO_2_, a midline thoracotomy was performed, and the heart was exposed. Peripheral blood was drawn from the right ventricle of the heart using a heparin-coated syringe. Purification of macrophages was performed using a ficoll density gradient (ficoll separation solution, SEROMED, density 1.077 g/ml). Blood (1.0 ml) was added to 4.0 ml PBS, mixed and layered on top of 5-ml ficoll solution. After centrifugation (800 ×g, 4°C, 24 min), the monocytes located in the separation layer were collected and washed twice with 8 ml PBS and re-centrifuged at 100 ×g for 5 min. The pelleted monocytes were collected and resuspended in 1 ml complete tissue culture medium (CTCM) and counted in a Neubauer chamber. Then the cells (5×10^5^) were placed in 25-well tissue culture plates containing RPMI plus 10% FBS+penicillin/streptomycin and incubated overnight[14].

### Microglia cells isolation

**Fig. 1:**
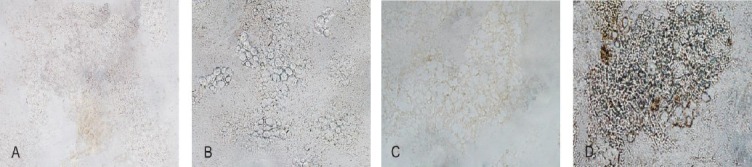
Immunocytochemistry. Immunophenotyping of microglial cells isolated from the lumbar part of rat’s spinal cord using immunocytochemical assay with OX42 monoclonal antibody against CR3/CD11 microglial cell marker. OX42 antibody reaction in free microglial cells isolated from A) sham-operated and C) CCI vehicle-treated rats. OX42 antibody reaction in microglial cells was isolated from B) sham-operated and D) CCI vehicle-treated rats. CCI vehicle-treated animals showed CR3/CD11 marker expression (magnification ⊆40).

Rats were sacrificed, their back were shaved and rinsed with 70% ethanol. Using a large scissors, the skin was removed, muscles surrounding spine were exposed and cut away from bottom to the top (neck) of backbone. The spinal cord was flushed and removed from the canal with 10 ml sterile Hank’s Balanced Salt solution. The lumbar part of the spinal cord was dissected and placed in a 60-mm dish containing cold culture media (DMEM). The whole dissection procedure was performed in less than 10 min. Under a dissecting microscope, the meninges were peeled away, and all the remaining attached dorsal and ventral roots were removed. The cord was transferred into a 35-mm dish full of trypsin/DNase (25% trypsin+50 µg/ml DNase) and split into two halves and sliced into pieces as thin as possible using two scalpel blades. Slices were incubated at 35ºC and shaken for 1 h and 30 min. The incubation was stopped by adding 2 ml heat-inactivated FBS to the dish. Using serum-coated pipette, tissues and fluids were transferred to a sterile centrifuge tube containing CTCM (90 ml DMEM+10 ml FBS+1 ml penicillin/streptomycin) and centrifuged at 4°C at 100 ×g for 10 min. The supernatant was removed, the pellet was resuspended by gentle agitation of the tube, and then 2 ml cold culture medium was added. Tissues were triturated on ice using gentle pipetting to dissociate neurons. Then a 20-µm nylon mesh was used to separate microglial cells in order to transfer them into an oak ridge tube containing 80% cold Percoll, sucrose 1.25 molar in 0.1 molar buffer phosphate and CTCM. The total volume of the cells was two times of the Percoll volume. Cells were centrifuged at 300 ×g for 45 min. Microglial cells, as a gray ring, were located between myelin layer at the top and the red blood cell layer at the bottom of the tube. The myelin layer was removed using a sterile pipette. Microglia cells were separated and transferred into a 15-ml tube filled with CTCM and then centrifuged at 100 ×g for 10 min. The supernatant was removed and CTCM was added to the pellet and again filtered. Microglial cells were plated at 1×10^6^ cells per well in 24-well tissue culture plates in DMEM containing 10% FBS and then incubated. After 24 h, tissue culture supernatants were collected and kept frozen for IL-6 assay[15]. Immunocytochemical assay was performed to distinguish resting microglia from activated cells using an OX-42 antibody against CR3/CD11b microglial cell receptors (Fig. 1).

### Immunocytochemical assay

Cells were seeded into Poly-D-Lysine coated glass coverslips and allowed to attach overnight. Then the cells were fixed using ethanol 95% plus glacial acetic acid 5%. After this step and during 4 hours, the cells were incubated with a specific biotinylated monoclonal antibody OX-42 (primary antibody to microglia CR3 complement receptor). Next, the cells were incubated with a secondary antibody (horseradish peroxidase streptavidine) for 2 hours. In the final step, diamino benzidene, as a chromogene, was applied for 30 minutes. The activated microglial cells, which were positive for OX-42 antibody, were stained and subsequently were visualized by a light microscope.

### IL-6 assay by ELISA

Cytokine levels in cell culture media of rat’s macrophage and microglia were measured using a commercially available ELISA specific for IL-6 (Biosource International, England). This kit provided a lower detection limit of <8 pg/ml.

### Statistical analysis

Tukey’s test (parametric) and two related samples followed by Wilcoxon test (non-parametric) were used to analyze the data. In all cases, *P*<0.05 was considered statistically significant.

## RESULTS

### Immunocytochemical assay

To distinguish resting microglia from activated cells, an OX-42 antibody against CR3/CD11b microglial cell receptors was used (Fig. 1).

### Response to mechanical allodynia (von Frey test)

**Fig. 2:**
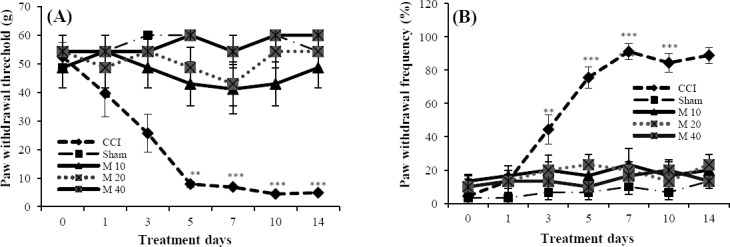
Pain behavior. Paw withdrawal threshold in response to von Frey filaments (A) and the frequency of paw withdrawal in response to acetone (B) before surgery and at different time points after surgery in CCI vehicle-treated, sham-operated and CCI minocycline-treated groups. Minocycline (M, 10, 20 and 40 mg/kg) was injected i.p. Data were analyzed using the analysis of variance (ANOVA) and were presented as means±S.E.M for six rats in each group. Asterisks (**P<0.01 and ***P<0.001) indicate a statistically significant difference between post surgery days and day 0.

All CCI saline-treated animals were strongly allodynic on the day 3 of post-ligation (*P*<0.01) compared to day 0 before surgery (control day). This effect was sustained until the end of the study (*P*<0.001). Sham-operated animals did not exhibit mechanical allodynia. Moreover, mechanical allodynia was not produced in CCI drug-treated animals (minocycline10, 20, and 40 mg/kg), when compared to the control day (Fig. 2A).

### Response to cold allodynia (acetone test)

CCI saline-treated rats exhibited pain behavior (*P*<0.01) at the day 3 of post surgery compared to day 0, which continued until the end of the study (*P*<0.001). However, this effect was not observed in sham-operated group during the experimental period. CCI drug-treated animals (minocycline 10, 20, and 40 mg/kg) did not show any pain behavior in comparison to day 0 (Fig. 2B).

### Cytokine assay in macrophage cell culture supernatant

**Fig. 3:**
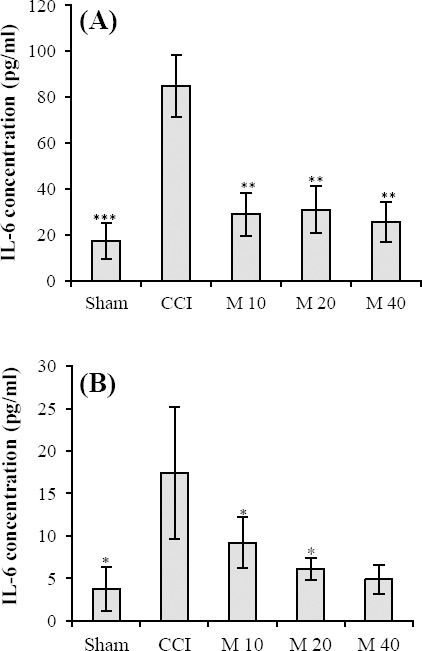
The IL-6 concentration in immune cells. IL-6 concentration of macrophage cells (A) and microglial cells (B) in CCI vehicle-treated, sham-operated and CCI minocycline-treated rats on day 14 post ligation. Data were analyzed using the analysis of variance (ANOVA) and were presented as means±S.E.M for six rats in each group. Asterisks (*P<0.05, **P<0.01 and ***P<0.001) indicate a statistically significant difference when compared to CCI vehicle-treated rats. M, minocycline (10, 20 and 40 mg/kg)

Protein analysis by ELISA revealed that minocycline 10, 20, and 40 mg/kg attenuated the production of IL-6 (*P*<0.01) compared to CCI saline-treated animals. In sham-operated animals there was no significant increase in the level of IL-6 (*P*<0.001) compared to CCI vehicle-treated animals (Fig. 3A).

### Cytokine assay in microglia cell culture supernatant

Protein analysis by ELISA indicated that minocycline 20 and 40 mg/kg decreased the level of IL-6 (*P*<0.05) compared to CCI saline-treated animals; however, minocycline 10 mg/kg did not reduce IL-6 level. In sham-operated animals, no increase was observed in the IL-6 level (*P*<0.05) compared to CCI vehicle-treated animals (Fig. 3B).

## DISCUSSION

In the present study, we evaluated the analgesic effects of minocycline in a CCI model of neuropathic pain in rat and measured the levels of secreted cytokine IL-6 from macrophage and microglial cells. It has recently been reported that neurons are not the only cell type involved in chronic pain states[17-20]. Evidence in the last decade has indicated that the pathogenesis of neuropathic pain not only changes the activity of neurons but also provides a close relation between neurons, inflammatory immune and glial cells, as well as immune cell-derived inflammatory cytokines and chemokines. Indeed, peripheral nerve injury provokes a reaction from the immune system (infiltration of inflammatory cells and the activation of resident immune cells), which has been observed at various anatomical locations, including the injured nerve, the dorsal root ganglia, the spinal cord and supraspinal sites associated with pain pathways[21].

Inflammatory mediators play a critical role in neuropathic pain generation. It seems that inflammatory mediators are generated at the site of injury, and glial cells in the spinal cord can also produce these mediators. In this regard, the main source of inflammatory mediators in the CNS is provided by microglia[2,5]. Among these mediators, cytokines such as TNF-α, IL-1, IL-6, and prostaglandins, which are produced by activated microglia, are involved in the neuronal damage and recruit immune cells into the CNS[22]. In this situation, macrophages and lymphocytes attracted by chemokines infiltrate into the injured peripheral nervous system and release these inflammatory cytokines[5]. Macrophages and microglia contribute to the secondary pathological and inflammatory responses through the release of TNF, IL-1, IL-6, and IL-10, which are involved in the activation of immune cells and neuropathic pain. Cytokines facilitate CNS inflammatory responses by inducing the expression of additional cytokines, chemokines, nitric oxide and reactive oxygen[23]. After nerve trauma and/or inflammation, pro-inflammatory cytokines and other mediators released by glia facilitate pain transmission. In this regard, microglia inhibitor could play a vital role in reducing the factors contributing to pain facilitation[24]. IL-6 released by macrophage can produce mechanical allodynia because infiltrating macrophages show high expression of this cytokine[25].

A comprehensive understanding of the pathogenesis of neuropathic pain is required to develop a better treatment. Microglia and astrocytes are emerging as possible additional players in the initiation and maintenance of inflammatory and neuropathic pain[20]. Currently, studies have attempted to find substances that inhibit the biosynthesis of pro-inflammatory cytokines. It has been reported that the drugs inhibiting glial activation can decrease cytokine release[11]. Blockade of inflammatory cytokines derived from various immune cells can prevent neuropathic pain after nerve injury. The increased levels of IL-6 in the spinal cord after peripheral nerve injury may participate in neuropathic pain development. In this condition, mechanical allodynia and thermal hyperalgesia are used as the indices of pain behavior[25,26]. IL-6 is a multifunctional cytokine with pro-inflammatory and regulatory effects produced by a variety of tissues, such as neural tissues and cell types like T cells, B cells, macrophages, fibroblasts, microglia and astrocytes. Injury of the sciatic nerve induces up-regulation of IL-6 and its receptor in the region of the lesion. IL-6 has been studied extensively in association with nerve damage and pain and is thought to play an essential role in the genesis of neuropathic pain[26]. Here, we evaluated the effect of minocycline on pain behavior. The results obtained in this study are consistent with the previous studies on the analgesic effects of minocycline in neuropathic pain[10,27]. Moreover, Padi and Kulkarni[28] evaluated the preventive effects of minocycline in reducing neuropathic pain in a CCI model. In another study, Burke *et al*.[29] demonstrated the pain-reducing effects of minocycline in a spinal nerve ligation model of neuropathic pain. Popiolek-Barczyk and coworkers[30] have found that in a CCI model, minocycline enhanced the effectiveness of nociceptin. In this regard, our data are in line with those mentioned studies.

In a mouse study, IL-6 was detected immediately after 2 hours after nerve transaction and lasted at least for 21 days. Three to 4 days following the nerve transaction, further cytokine secretion is induced by macrophages, which are recruited to the area. It is well-known that IL-6 and IL-1β have an important role in neuropathic pain[31]. Evidence indicates that neuroimmune activation is associated with the generation and maintenance of behavioral changes during neuropathic pain when neurons and glia produce a variety of inflammatory cytokines, including TNF-α, IL-1β, IL-6 and IL-10. These cytokines can increase the nociceptive activity and also severe and persistent neuropathic pain[32]. We observed a decreased level of IL-6 in cell culture media of macrophage and microglial cells. These data are consistent with the previous works on cytokine assay in these immune cells in an injury model of sciatic nerve[31,33].

Minocycline has demonstrated neuroprotective effects in experimental models of CNS trauma, stroke and spinal cord injury[34]. Minocycline has also indicated to reduce the cytokine levels (IL-1β, TNF-α, and IL-6), which promoted neuro-inflammation and decreased pain behavior in a CCI model of neuropathic pain[24]. Although microglia may be a target of minocycline action, it is possible that this drug has activity on other immune cell subsets, such as T cells, which have important roles in different CNS disorders. Direct interaction of T lymphocytes with microglia through cell-cell contact mechanisms up-regulates the production of pro- and anti-inflammatory cytokines[35]. A worth noting aspect of minocycline actions is the increased production of IL-10 in the microglia-T cell coculture when the T cells are pretreated with the higher concentrations of minocycline. IL-10 is an immunoregulatory cytokine that can inhibit microglia activation[35].

In conclusion, we found that minocycline, as a microglia inhibitor, can provide neuroprotective effects by reducing exaggerated pain due to the CCI model of neuropathic pain and reducing the inflammatory process by decreasing IL-6 concentration released by central and peripheral immune cells (microglia and macrophages) involved in inflammatory process in neuropathic pain. More searches are needed to find the exact mechanisms of minocycline in neuropathic pain and to discover new analgesic drugs to alleviate pain regarding the contribution of immune cells in neuropathic pain condition.
